# Paeoniflorin Ameliorates Experimental Autoimmune Encephalomyelitis via Inhibition of Dendritic Cell Function and Th17 Cell Differentiation

**DOI:** 10.1038/srep41887

**Published:** 2017-02-06

**Authors:** Han Zhang, Yuanyuan Qi, Yuanyang Yuan, Li Cai, Haiyan Xu, Lili Zhang, Bing Su, Hong Nie

**Affiliations:** 1 Shanghai Institute of Immunology, Department of Immunology and Microbiology, Shanghai Jiao Tong University School of Medicine, Shanghai, PR China; 2 Department of Clinical Immunology, Shanghai Ninth People’s Hospital, Shanghai Jiao Tong University School of Medicine, Shanghai, PR China

## Abstract

Paeoniflorin (PF) is a monoterpene glycoside and exhibits multiple effects, including anti-inflammation and immunoregulation. To date, the effect of PF on multiple sclerosis (MS) has not been investigated. In this study, we investigated the effect of PF in experimental autoimmune encephalomyelitis (EAE), an animal model for MS. After administered with PF, the onset and clinical symptoms of EAE mice were significantly ameliorated, and the number of Th17 cells infiltrated in central nervous system (CNS) and spleen was also dramatically decreased. Instead of inhibiting the differentiation of Th17 cells directly, PF influenced Th17 cells via suppressing the expression of costimulatory molecules and the production of interlukin-6 (IL-6) of dendritic cells (DCs) *in vivo* and *in vitro*, which may be attributable to the inhibition of IKK/NF-κB and JNK signaling pathway. When naïve CD4^+^ T cells were co-cultured with PF-treated dendritic cells under Th17-polarizing condition, the percentage of Th17 cells and the phosphorylation of STAT3 were decreased, as well as the mRNA levels of IL-17, RORα, and RORγt. Our study provided insights into the role of PF as a unique therapeutic agent for the treatment of multiple sclerosis and illustrated the underlying mechanism of PF from a new perspective.

Multiple sclerosis (MS) is an autoimmune disease characterized by chronic inflammation and demyelination in the central nervous system (CNS). Experimental autoimmune encephalomyelitis (EAE) is a well-established animal model for studying the underlying pathogenesis and developing new therapies for MS^1^,^2^. The molecular mechanisms of the disease are complex, and it is widely accepted that autoreactive Th cells are critically involved in the development of this disease^3^,^4^. Numerous reports have well studied the significant role of Th1 cells in EAE, however, abundant evidence demonstrate that Th17 cells are the main effector cells in the pathogenesis of EAE^5^,^6^,^7^.

Antigen-presenting cells (APCs) play a critical role in the initiation and development of adaptive immune responses. Dendritic cells (DCs), as a professional APC, are uniquely capable of presenting antigenic peptides to naïve T cells for clonal expansion^8^,^9^,^10^. Bone marrow-derived DCs (BMDCs) located at peripheral tissue are immature DCs (iDCs) that differentiate into mature DCs (mDCs) when stimulated with antigen. Then the latter migrate to lymphoid tissue and participate in immune response. The phenotypes of DCs at different differentiation stages are diverse, including MHCII and costimulatory molecules CD80, CD86, and CD40, which interact with corresponding ligands to provide signals for activating T cells^11^,^12^. In addition, pro-inflammatory cytokines IL-12 and IL-6 secreted by DCs are crucial in promoting the differentiation of Th1 cells and Th17 cells, respectively^13^,^14^,^15^,^16^. Given the important role they play in the immune system, DCs are a potential target for the treatment of immune-mediated inflammatory diseases.

As a mixture of various bioactive components, total glucosides of paeony (TGP) are extracted from the roots of Paeonia lactiflora and have been widely used as an anti-inflammatory remedy in the therapy for autoimmune diseases in Chinese medicine. Recent studies have explored the effects of TGP on rheumatoid arthritis^17^, experimental diabetes^18^ and experimental liver injury^19^. As the principal bioactive component of TGP, Paeoniflorin (PF) is a monoterpene glycoside (C_23_H_28_O_11_) and accounted for more than 90% of TGP^20^. It has been reported that PF exhibits multiple effects, such as anti-inflammation, immunoregulation, anti-arthritis, anti-hepatitis, pain-relieving, neuroprotection, and anti-hyperglycemia^17^,^21^,^22^,^23^. To date, the therapeutic effect of PF on MS has not been investigated. In this study, we explored the potential treatment efficacy of PF on mouse MS model and revealed the underlying mechanism by investigating its influence on Th cells and DCs.

## Results

### PF ameliorated the symptoms and delayed the onset of EAE

**Figure 1:**
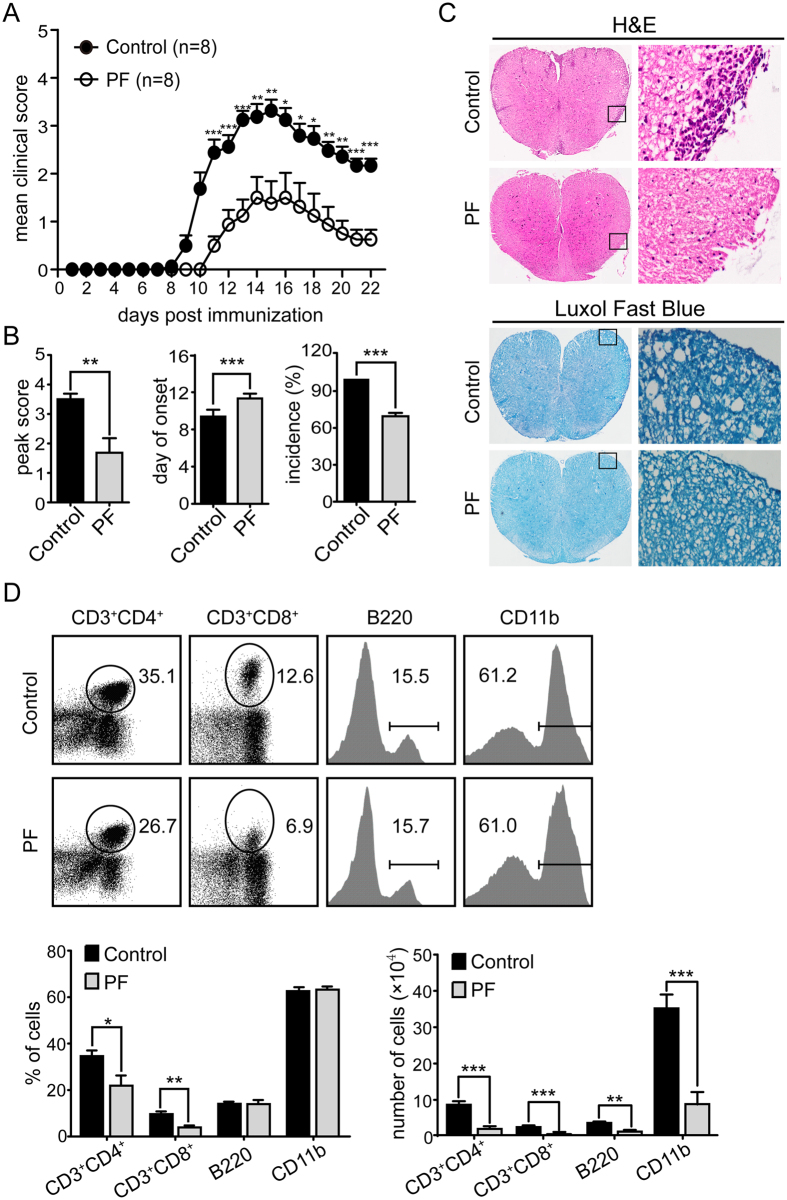
PF ameliorated EAE in mice. EAE was induced in C57BL/6 mice by immunizing with MOG_35–55_ peptide. Mice were injected i.p. daily with PBS (Control) or PF (5 mg/kg) started from day 4 before EAE induction and each group consisted of 8 mice. (**A**) Clinical scores were recorded daily. (**B**) The cumulative mean peak score, average day of onset and disease incidence of PF-treated or control EAE mice were represented. (**C**) Representative H&E and luxol fast blue staining of spinal cords sections obtained from PF-treated or control EAE mice at day 15 post immunization (left panel, ×40; right panel, ×200). (**D**) Percent of CD3^+^CD4^+^, CD3^+^CD8^+^, B220^+^ (in lymphocyte gate) and CD11b^+^ (in total MNC gate) cells in MNCs obtained from CNS in PF-treated or control EAE mice. Data are representative of three independent experiments with eight mice per group. Values were expressed as mean ± SEM. *p < 0.05; **p < 0.01; ***p < 0.001.

To evaluate the anti-inflammatory properties of PF in MS, we initially investigated the effect of systemic administration of PF on EAE induced by MOG_35–55_ peptide. PF administration (5 mg/kg/d) starting from day 4 before immunization and continued daily thereafter. As illustrated in Fig. 1A, PF-treatment mice showed a significant decrease in mean clinical score compared with control group. Furthermore, the treatment of PF also resulted in significantly reduced mean peak score and disease incidence, and delayed disease onset compared to control group (Fig. 1B). These observations prove the protective efficacy of PF against MS *in vivo*.

### PF treatment alleviated CNS pathological manifestations

Inflammatory cell infiltration and demyelination are the major pathological manifestations in the CNS of EAE mice. To further confirm the anti-inflammatory effect of PF, H&E and Luxol fast blue staining were performed to analyze the neuropathological changes of spinal cord tissues. Compared to control group, PF administration remarkably attenuated the degree of infiltrated inflammatory cells and demyelination in EAE mice (Fig. 1C). The number and percentage of infiltrated MNCs in CNS of PF-treated and control EAE mice was analyzed by flow cytometry. The expression of CD3, CD4, CD8, B220, and CD11b on MNCs purified from CNS was applied for distinguishing infiltrated T-cell, B-cell, and macrophages/microglia, respectively. PF treatment resulted in significantly reduced percentages of CD4^+^ and CD8^+^ T cells. More importantly, the absolute numbers of these cells were remarkably decreased in PF-treated EAE mice compared with that in control EAE mice (Fig. 1D). Taken together, these results indicate that PF obviously ameliorates the inflammatory reaction of EAE.

### Amelioration of EAE by PF treatment was correlated with decreased Th17 cells

**Figure 2:**
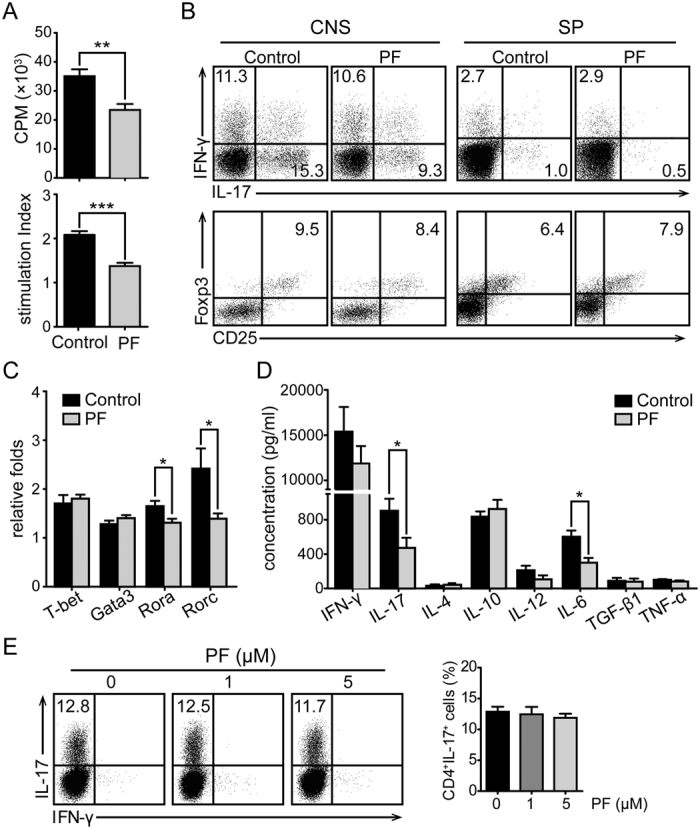
Encephalitogenic T cell response and cytokine profile in response to MOG_35–55_ peptide in EAE mice treated with PF. Splenocytes or CNS MNCs from PF-treated or control EAE mice at day 15 post-immunization were isolated. (**A**) CD4^+^ T cells sorted from splenocytes were co-cultured with irradiated splenocytes isolated from naïve mice with the stimulation of MOG_35–55_ peptide and examined for proliferation. The stimulation index (SI) of CD4^+^ T cells was calculated as follows: SI = (the cpm value of co-cultured cells - the cpm value of irradiated splenocytes)/the cpm value of CD4^+^ T cells. (**B**) The percentage of Th1, Th17, and Treg cells in CD4^+^ T cells were measured by flow cytometry through intracellular staining of IL-17, IFN-γ, and Foxp3. (**C**) mRNA expression of T-bet, Gata3, RORα, and RORγt in CD4^+^ T cells were analyzed by real-time PCR. (**D**) Splenocytes were stimulated with MOG_35–55_ for 48 h, and supernatants were collected to detect the concentrations of inflammatory cytokines. Data are representative of three independent experiments with eight mice per group. Values were expressed as mean ± SEM. *p < 0.05; **p < 0.01; ***p < 0.001. (**E**) Naïve CD4^+^ T (CD4^+^CD62L^+^) cells were induced differentiation into Th17 cells in different concentrations of PF. The percentage of Th17 cells in CD4 gate was measured. Data are representative of at least three independent experiments.

To investigate the underlying mechanism for anti-inflammatory property of PF, we first examined the profile of CD4^+^ T cells in EAE mice. As illustrated in Fig. 2A, when stimulated with MOG_35–55_ peptide *ex vivo*, the proliferation of CD4^+^ T cells purified from PF-treated EAE mice was markedly dampened. Given the crucial role of autoreactive pathogenic Th1 and Th17 cells in the pathogenesis of EAE, the Th cell subsets of MNCs derived from CNS and spleen of EAE mice were analyzed by flow cytometry. Specifically, the percentage of Th17 (CD4^+^IL-17^+^) cells both in the spleen and CNS was significantly reduced after PF treatment, whereas the relative numbers of Th1 (CD4^+^IFN-γ^+^) and Treg (CD4^+^CD25^+^Foxp3^+^) cells were not influenced obviously (Fig. 2B). Consistently, RORγt and RORα, key transcriptional factors of Th17 cells, were obviously decreased in CD4^+^ T cells derived from PF-treated EAE mice (Fig. 2C). Moreover, levels of IL-17 and IL-6 (Th17 cytokines) were remarkably decreased by PF treatment (Fig. 2D). These data suggest that PF ameliorates inflammation of EAE via decreasing the percentage of Th17 cells *in vivo*.

To further delineate whether PF suppresses inflammation through influencing Th17 cells directly, we observed the effect of PF on the differentiation of Th17 cells *in vitro*. As shown in Fig. 2E, no obvious changes were detected in Th17 cell differentiation at different concentrations of PF. The result indicates that PF do not directly work on Th17 cells.

### The expression of costimulatory molecules, MHC II, and the secretion of IL-6 by DCs were inhibited by PF *in vivo* and *in vitro*

**Figure 3:**
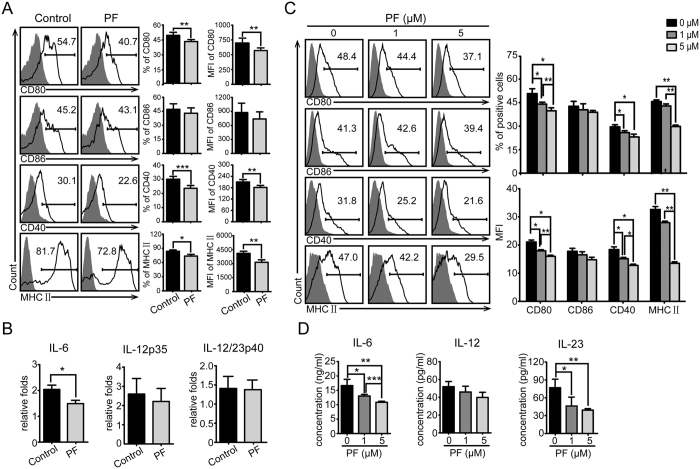
Effect of PF on CD11c^+^DCs and BMDCs. (**A**) Splenocytes from PF-treated or control EAE mice were analyzed for the expression of CD80, CD86, CD40, and MHC II in CD11c^+^ gate by flow cytometry. (**B**) mRNA levels of IL-6, IL-12p35, and IL-12/IL-23p40 in CD11c^+^DC were analyzed by real-time PCR. Data are representative of three independent experiments with eight mice per group. Values were expressed as mean ± SEM. *p < 0.05; **p < 0.01, ***p < 0.001. (**C**) Bone marrow cells were stimulated by GM-CSF and IL-4 for 5 days to induce BMDCs in different concentrations of PF, and the expression of indicated markers were analyzed by flow cytometry. (**D**) The production of cytokines in the culture supernatants from BMDCs stimulated with LPS was examined by ELISA. Data are representative of at least three independent experiments. Values were expressed as mean ± SEM. *p < 0.05; **p < 0.01, ***p < 0.001.

APCs are critically involved in the initiation of adaptive immunity. However, only DCs could prime naïve T cells for initiating antigen-specific CD4 T cell immune responses. Accumulating evidence has demonstrated that control of DCs can influence the development of autoimmune and inflammatory diseases^24^,^25^. Skarica *et al*. reported a significant reduction of Th1 and Th17 immune responses in EAE by inhibiting APCs^26^. Thus, it is crucial to further study whether PF could modulate DC functions. To this end, we first analyzed the phenotype of CD11c^+^DCs. Splenocytes derived from PF-treated and control EAE mice were analyzed for the expression of costimulatory molecules and MHC II in CD11c^+^ gate by flow cytometry. As shown in Fig. 3A, the expression levels of CD80, CD40, and MHC II were decreased by PF treatment, whereas the surface expression of CD86 was not altered. Then we detected the effect of PF on the production of cytokines from DCs. The real-time PCR results showed that PF inhibited the expression of IL-6 in CD11c^+^DCs, whereas IL-12p35 and IL-12/23p40 were not significantly affected (Fig. 3B). To verify the inhibiting effect of PF on DCs, bone marrow cells were induced for BMDC differentiation at different concentrations of PF *in vitro*. In consistent, the expression of surface markers was inhibited by PF in a dose-dependent manner (Fig. 3C). Moreover, IL-6 and IL-23 production of BMDCs was decreased by PF *in vitro* (Fig. 3D), which was consistent with the observed effect of PF *in vivo*. These results suggest that PF could suppress DC function and thus lead to ameliorating the inflammatory reaction of EAE.

### PF impaired Th17 cell differentiation through inhibiting IL-6 production by DCs

**Figure 4:**
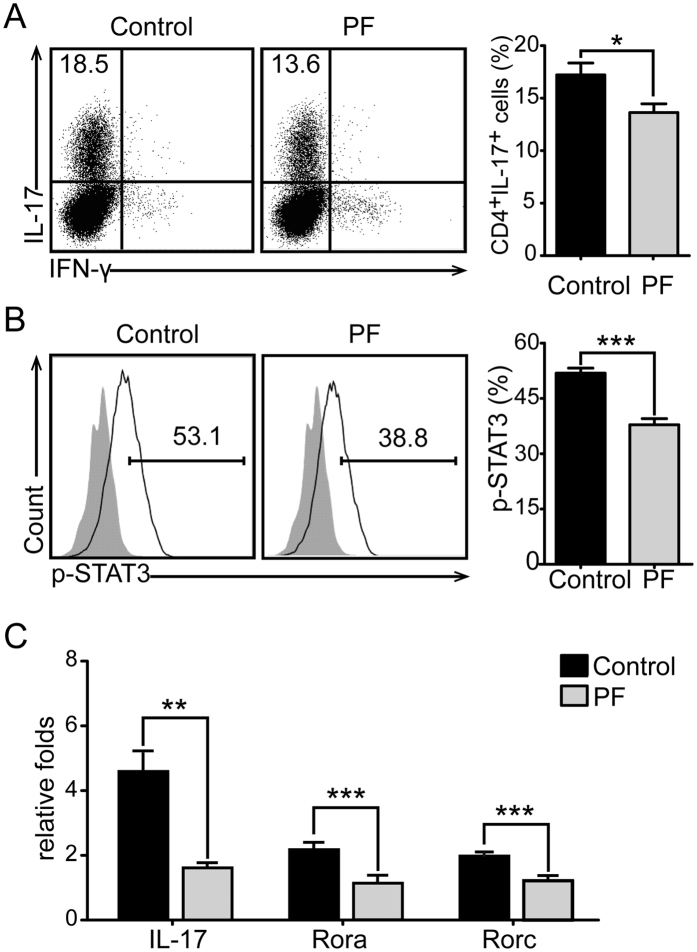
PF inhibited IL-6 production of CD11c^+^DCs and impaired Th17 cell differentiation. Naïve CD4^+^ T cells were co-cultured with CD11c^+^DCs from PF-treated or control EAE mice spleen for 3 days under Th17-polarizing conditions. The percentage of Th17 cells (**A**) and the phosphorylation of STAT3 (**B**) were analyzed by flow cytometry in CD4 gate. (**C**) mRNA levels of IL-17, RORα, and RORγt were analyzed by real-time PCR. Data are representative of three independent experiments. Values were expressed as mean ± SEM. *p < 0.05; **p < 0.01; ***p < 0.001.

**Figure 5:**
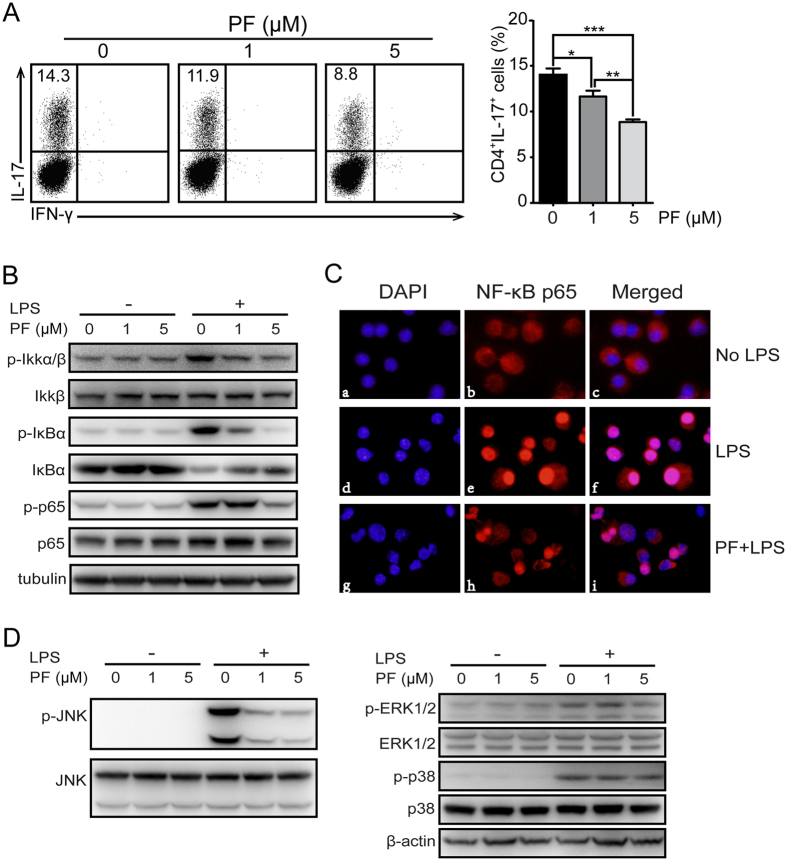
PF inhibited IL-6 production of BMDCs and impaired Th17 cell differentiation via suppressing IKK/NF-κB and MAPK signaling pathway *in vitro*. Bone marrow cells from naïve mice were stimulated by GM-CSF and IL-4 to induce DC differentiation in the presence or absence of the indicated concentrations of PF for 5 days, and followed by the stimulation of LPS. (**A**) The above DCs were co-cultured with naïve CD4^+^ T cells for Th17 cell differentiation. The percentage of Th17 cells were analyzed by flow cytometry. (**B**) The protein expression of IKK/NF-κB signaling pathway was analyzed by immunoblot assay. Tubulin served as control. (**C**) The nucleus translocation of NF-κB-p65 was monitored by fluorescence microscopy (400×). Nuclei were stained in blue; the NF-κB p65 was stained in red. (**D**) Protein expression of MAPK signaling pathway was detected by immunoblot assay. β-actin was detected as control. Data are representative of three independent experiments. Values were expressed as mean ± SEM. *p < 0.05; **p < 0.01; ***p < 0.001.

It has been confirmed that cytokines derived from DCs play a controlling role in Th cell subset differentiation. Because the inhibition of PF on Th17 cell differentiation was only observed *in vivo* in this study, but not *in vitro*, we assumed that PF might affect Th17 cell polarization indirectly by regulating DCs-derived cytokines. Therefore, we examined whether the reduction of CD11c^+^ DCs-derived IL-6 would affect Th17 cell differentiation. For this purpose, naïve CD4^+^ (CD4^+^CD62L^+^) T cells were co-cultured with CD11c^+^DCs sorted from PF-treated and control EAE mice under Th17-polarizing conditions to measure Th17 cell differentiation and the related events. IL-6 was known to play critical roles in Th17 cell differentiation^27^, and STAT3, which is activated by IL-6, is a key transcription factors for this differentiation^28^. As shown in our data, both the percentage of Th17 cells (Fig. 4A) and phosphorylation level of STAT3 (Fig. 4B) were markedly reduced in CD4^+^ T cells that cocultured with PF treatment CD11c^+^DCs. mRNA levels of IL-17, RORα and RORγt were decreased consistently (Fig. 4C). Furthermore, the effect of PF-treated BMDCs on Th17 cell differentiation was measured by coculturing with the above naïve CD4^+^ T cells under Th17-polarizing conditions. As illustrated in Fig. 5A, the differentiation of Th17 cells was obviously impaired in PF dose-dependent manner. These data demonstrates that PF inhibits Th17 cell differentiation by decreasing the production of DCs-derived IL-6.

### PF inhibited DC function via suppressing IKK/NF-κB and JNK activity

Previous studies have confirmed the crucial role of IKK/NF-κB activation in the function of DCs including costimulatory molecules expression and inflammatory cytokines production^29^,^30^. Once IKK/NF-κB pathway is activated, IκB is activated by phosphorylated IKK and then degraded, followed by activated NF-κB p65 translocating into the nucleus where it regulates target gene expression^31^,^32^. Therefore, the protein expression of IKK/NF-κB signaling pathway and NF-κB p65 nuclear translocation were detected by immunoblot and immunofluorescence assay, respectively. As present in our data, the protein expression of pho-Ikkα/β, pho-IκBα and pho-NF-κB p65 was reduced by PF treatment, and the degradation of IκBα was also decreased (Fig. 5B). In accordance with the above results, the nuclear translocation of NF-κB p65 was significantly decreased in PF-treated BMDCs (Fig. 5C). Since mitogen activated protein kinases (MAPKs) pathway plays important roles in inflammatory response^33^, the effects of PF treatment on MAPK pathway, including extracellular signal-regulated kinase (ERK), c-Jun N-terminal kinase (JNK) as well as p38 MAPK, were explored. Immunoblot data showed that the protein expression of p-JNK was substantially decreased with elevating in the concentration of PF, whereas the expression of p-ERK and p-p38 was not affected obviously (Fig. 5D). These results suggest that PF suppress DC function through inhibiting the activation of IKK/NF-κB and JNK.

## Discussion

Currently, it has been widely accepted that multiple elements of immune system and CNS play crucial roles in the pathogenesis and development of MS and EAE. The progression of this autoimmune disease covers numerous steps that can be divided into two stages: early phase including initiating and establishing the autoimmune response to myelin components in the periphery immune system, later phase associated with the destructive inflammatory responses in CNS including demyelination and axonal damage^34^. In the present study, we demonstrated that PF provided a significantly therapeutic effect on MOG_35–55_-induced EAE. As a result, PF treatment ameliorated the clinical signs, delayed the onset and reduced the incidence of EAE. The therapeutic effect of PF was associated with a significant reduction in inflammatory cell infiltration and the subsequent demyelination of CNS.

Autoreactive Th1 (producing IFN-γ) and Th17 (producing IL-17) cells are primed and developed outside the CNS, once activated by myelin components, they enter into CNS and then further recruit other immune cells to activate resident macrophages/microglia, thus leading to the damage of CNS structures and resulting in progressive paralysis^2^. Adoptively transferring myelin-reactive Th1 and Th17 cells to naïve mice also can induce clinical signs of EAE. In addition, levels of IFN-γ and IL-17 are markedly elevated in MS patients and EAE mice^35^. Therefore, as drivers of autoimmune response, pathogenic Th1 and Th17 cells are considered as the principal criminal in MS and EAE^36^. Our data demonstrated that treatment with PF markedly decreased Th17 cells percentage in the spleen and CNS at the peak period, whereas Th1 and Treg cells were not affected. Considering the result that PF delayed the onset of EAE, we proposed that PF may mainly inhibit the immune response in early stage, primarily through affecting Th17 cell polarization that has been thought as key factor in eliciting EAE^37^. However, inconsistent with the results *in vivo*, Th17 cells differentiation *in vitro* was not altered. These results suggested that PF suppressed Th17 cells differentiation via indirect means *in vivo*, resulting in the amelioration of EAE.

The activation of myelin-reactive Th cells is the predominant event in the progression of EAE. In addition, APCs also play an important role in the pathogenies and development of EAE. As professional APCs, DCs are uniquely able to directly activate naïve T cells and initiate autoimmune response in the periphery. Activation and differentiation of myelin-reactive Th cells are directed by DC through three signals. After being processed by DCs, antigen is presented to T cell recognition through MHCII, providing the first activation signal. The costimulatory molecules of CD80, CD86 and CD40 on the surface of DCs provide a second signal of T cell proliferation. The third signal is pro-inflammatory cytokines secreted by DCs, which determined the differentiation of activated Th cells^16^. Our data showed that PF significantly affected the early stage of immune response — Th cell activation. To further explore the mechanism of PF-induced amelioration of EAE, we next investigated the effect of PF on CD11c^+^DC in mice spleen *in vivo* and BMDCs *in vitro* which share a set of common features as naturally derived DCs^35^. Our results showed that PF reduced the expression of costimulatory molecule CD80 and CD40 as well as MHCII *in vivo*, in a dose dependent manner, whereas CD86 were not affected. Consistent with the data *in vivo*, the expression of CD80, CD40 and MHCII on BMDCs was significantly decreased. In this study, we demonstrated for the first time that PF decreased the CD40 expression in CD11c^+^DCs and BMDCs. A previous study has demonstrated the significantly decreased differentiation of Th17 rather than Th1 in CD40-dificient mice^38^, and another study detailed a necessary of CD40 expression on DC for inducing Th17 differentiation *in vitro* and *in vivo*^16^. Therefore, our results suggested that treatment with PF markedly decreased the presence of Th17 cells through suppressing the surface molecule expression on DCs.

Besides surface molecule expression on DCs, DC-derived cytokines are also predominant elements for inducing Th17 cell differentiation. Previous reports have described the predominant role of IL-6 and TGF-β in mouse Th17 cell induction^39^,^40^,^41^ and IL-23 in maintaining and expanding the developing Th17 population^38^,^42^. However, TGF-β involves in both Th17 and Treg cell differentiation, promoting pro-inflammatory and anti-inflammatory response, respectively^43^. The presence of IL-6 seems to be a key determinant of Th17 polarization. IL-6 deficient mice are completely resistant to EAE induction^44^, and IL-6R blockade can prevent the development of EAE by inhibiting the number of MOG-active CD4^+^ T and CD8^+^ T cells in CNS and Th17 cells in peripheral lymphoid tissue^45^. However, neither pathogenic T cells nor CNS-resident cells are required to produce IL-6 in EAE. Instead, the requirement for IL-6 is entirely controlled by DC-derived IL-6^16^,^46^. These studies revealed that Th17 induction is critically dependent upon IL-6 derived from the initiated DCs. In this study, we found that PF treatment reduced IL-6 expression in CD11c^+^DCs from EAE mice *in vivo* and in BMDCs *in vitro*. Our results are consistent with other studies indicating that PF treatment reduced IL-6 production of macrophages^47^,^48^ and decreased serum IL-6 levels in autoimmune hepatitis mice^49^. Moreover, we found that Th17 polarization was reduced when either CD11c^+^DCs from PF-treated EAE mice or PF-treated BMDCs were used as the source of IL-6. Our results indicated that PF inhibited IL-6 production of DCs, and thus resulted in decreased Th17 polarization.

The major role of IKK/NF-κB signaling pathway in the pathogenesis of MS/EAE is well reported by various findings^50^,^51^,^52^. Previous studies showed that IKK/NF-κB activation contributed to LPS-mediated surface molecule up-regulation and induced transcription of pro-inflammatory target genes^30^. Our study showed that PF suppressed the LPS mediated activation of IKK/NF-κB signaling pathway in BMDCs by inhibiting the phosphorylation of IKKα/β, IκBα and NF-κB p65. Less amount of nuclear NF-κB p65 in PF-treated BMDCs also proved the inhibition of PF on IKK/NF-κB activation. In addition to the IKK/NF-kB pathway, LPS stimulation has been shown to activate MAPK signaling pathway. It has been demonstrated that three MAPKs, including ERK, JNK, and p38, are involved in the development of EAE^53^,^54^,^55^. In the present study, we illustrated that PF could significantly suppress the phosphorylation of JNK in BMDCs, suggesting that PF could suppress JNK cascades.

In summary, we demonstrated that PF treatment can effectively improve the clinical symptoms and delay the disease onset in EAE. In our present study, PF decreased IL-6 production and down- regulated costimulatory molecule expression in DCs via impairing the IKK/NF-κB and JNK signal activation, thus leading to decreased inflammatory response induced by Th17 cells. Our findings support the potential therapeutic effect of PF for MS.

## Materials and Methods

### Mice and reagents

Male C57BL/6 mice (6 weeks old) were obtained from the Shanghai Laboratory Animal Center, Chinese Academy of Science. Mice were housed under pathogen-free conditions in Shanghai Jiao Tong University School of Medicine. All experimental procedures were approved by the Animal Care and Use Committee of Shanghai Jiao Tong University School of Medicine and performed according to the guidelines. PF was purchased from Yilin Biological Technology Co., Ltd. (Shanghai, China) and had a purity of 98%. PF was dissolved in sterile phosphate buffered saline (PBS) to provide stock solution.

### Induction and assessment of EAE

To induce EAE, mice were immunized subcutaneously with 300 μg MOG_35–55_ peptide (MEVGWYRSPFSRVVHLYRNGK; GL Biochem, Shanghai, China) emulsified in total 100 μl CFA containing 5 mg/ml heat-killed H37Ra strain of *Mycobacterium tuberculosis* (Difco, 231141, MI, USA). On the day of immunization and 2 days later, the mice were administered *i.v.* with 200 ng pertussis toxin (Merck, 516562, CA, USA) dissolved in PBS. Mice were observed daily and scored for disease severity on a scale of 0–5: 0, no clinical sign; 1, limp tail; 2, one hindlimb paralysis; 3, bilateral hindlimb paralysis; 4, hindlimb and forelimb paralysis; 5, moribund or dead. PF was administered i.p. at 100 μg/mouse daily starting from 4d before immunization, and equal volume of PBS was served as control.

### Histopathological analysis

Spinal cords from PF-treated and control EAE mice were immediately immersed in 4% paraformaldehyde for fixation. After 2 days later, the specimen was embedded in paraffin for sectioning. The paraffin sections (5 μm thickness) were stained with H&E and luxol fast blue for assessing the inflammatory cell infiltration and demyelination, respectively.

### Isolation of Mononuclear cells

To isolate the infiltrating mononuclear cells (MNCs) from spinal cord and brain (referred to as CNS hereafter), cardiac perfusion with PBS was first performed in EAE mice to eliminate the peripheral blood cells. The dissociated CNS tissue was gently grinded to prepare for cell suspension. MNCs from CNS were isolated using Percoll (GE Healthcare, 17–0891–02, MD, USA) density gradient (37% and 70%) centrifugation.

### MOG-specific CD4^+^ T cells response *ex vivo*

Spleens from PF-treated and control EAE mice were removed and prepared for single-cell suspensions. CD4^+^ T cells were magnetically sorted by CD4 (L3T4) MicroBeads (Miltenyi biotech, 130–049–201, CA, USA) according to the manufacturer’s instruction (the purity >95%). Purified CD4^+^ T cells (2 × 10^5^) were cultured in triplicate with MOG_35–55_ peptide (20 μg/ml), and 2 × 10^5^ γ-ray irradiated splenocytes isolated from naïve mice were used as APCs. The cells were cultured at 37 °C in 5% CO_2_ for 72 h in RPMI-1640 (Gibco, 11875–093, CA, USA) medium supplemented with 10% fetal bovine serum (Gibco, 10099–141), 100 IU/ml penicillin, 100 μg/ml streptomycin, 2 mM L-glutamine (Gibco, 25030–081), 10 mM Hepes (Gibco, 15630–080), and 55 mM β-mercaptoethanol (Gibco, 21985–023). 0.5 μCi ^3^H-thymidine (Institute of Shanghai atomic energy, Shanghai, China) was added to cells at the last 16 h of culture. ^3^H-thymidine incorporation was detected as cpm using a Betaplate counter (PerkinElmer, MA, USA).

### Th cell differentiation *in vitro*

CD4^+^CD62L^**+**^T Cell Isolation Kit II (Miltenyi Biotech, 130–093–227) was used to sort naïve CD4^**+**^ T cells in spleen isolated from naïve mice. Purified naïve CD4^**+**^ T cells (1.5 × 10^5^ per well) were stimulated with plate-bound anti-CD3 Ab (1 μg/ml; BD biosciences, 553057, 145–2C11, CA, USA) and soluble anti-CD28 Ab (1 μg/ml; BD biosciences, 553294, 37.51) under Th17-polarizing condition in different concentrations of PF (0, 1, and 5 μM) and cultured for 3 days to induce Th17 cell differentiation. Th17-polarizing condition was as follows: 10 ng/ml IL-6 (R&D System, 406-ML-005, MN, USA), 1 ng/ml TGF-β (PeproTech, 100-21C, NJ, USA), 50 ng/ml IL-23 (PeproTech, 200-23), 10 μg/ml anti-IFN-γ (eBioscience, 16-7311-85, CA, USA), and 10 μg/ml anti-IL-4 (eBioscience, 16-7041-85).

### Isolation of DCs from mouse spleen

For isolation of spleen DCs, spleens were cut into small pieces and incubated for 1 h at 37 °C with 1 mg/ml collagenase D (Roche, 11088866001, CA, USA) and 0.02 mg/ml DNase I (Roche, 11284932001) in RPMI-1640. Single cell suspension was prepared by grinding the small pieces through a 70 μm cell strainer. Then cells were blocked by FcR Blocking Reagent (eBioscience, 14-0161-85, 93). CD11c^+^ cells were magnetically sorted by CD11c MicroBeads (Miltenyi Biotech, 130-097-059) according to the manufacturer’s instruction.

### Bone marrow-derived DCs generation

Bone marrow-derived DCs (BMDCs) were generated from mice bone marrow cells as described previously^56^. Briefly, the bone marrow was isolated from femurs and red blood cells were lysed. The bone marrow cells were incubated with 10 ng/ml GM-CSF and IL-4 (PeproTech, 315-03 and 214–14, respectively) for 5 days in different concentrations of PF (0, 1, and 5 μM) to obtain BMDCs. To induce cytokine secretion or Th17-polarizing, BMDCs with or without PF treatment were stimulated with 100 ng/ml LPS (Sigma-Aldrich, L6529, MO, USA) for 18 h.

### Flow cytomerty

For surface markers, cells were stained with fluorescent-conjugated antibodies (Abs) or isotype control Abs at the recommended dilution for 30 min in 4 °C away from light. MNCs purified from CNS were stained with Abs to CD3 (eBioscience, 11–0031, 145–2C11), CD4 (eBioscience, 48–0041, GK1.5), CD8 (eBioscience, 25–0081, 53–6.7), B220 (eBioscience, 12–0452, RA3–6B2), and CD11b (eBioscience, 17–0012, M1/70), while splenocytes and BMDCs were stained with CD11c (eBioscience, 12–0114, N418), CD80 (eBioscience, 11–0801, 16–10A1), CD86 (eBioscience, 11–0862, GL1), CD40 (eBioscience, 17–0401, 1C10), and MHC II (eBioscience, 125–5321, M5/114.15.2) Abs. For intracellular cytokine staining, MNCs from CNS, splenocytes, and differentiated Th cells were stimulated with Cell Stimulation Cocktail (eBioscience, 00–4975) for 5 h. Cells were first stained with anti-CD4 Ab (eBioscience, 11–0041, GK1.5). Then the cells were washed, fixed, and permeabilized with Cytofix/Cytoperm buffer (BD Biosciences, 554722), following intracellular cytokine staining with Abs against IL-17 (eBioscience, 12–7177, eBio17B7) and IFN-γ (eBioscience, 17–7311, XMG1.2). To stain CD4^+^CD25^+^Foxp3^+^Treg, cell surface staining with CD4 (eBioscience, 11–0041, GK1.5) and CD25 (eBioscience, 12–0251, PC61.5) Abs was performed before fixed and permeabilized with Foxp3 Permeabilization buffer (eBioscience, 00–5223–56), and intranuclear Foxp3 (eBioscience, 17–5773, FJK-16 s) staining was followed. To stain phosphorylated STAT3, differentiated Th cells were fixed with 2% paraformaldehyde for 10 min at 37 °C and followed by permeabilization with 90% methanol for 30 min on ice. Then the cells stained with CD4 (eBioscience, 11–0041, GK1.5) and p-STAT3 (BD biosciences, 612569, 4/P-STAT3) Abs. Flow cytometric data were acquired on BD FACS CantoII, and analyzed using Flowjo software.

### Measurement of cytokines

Splenocyte (1 × 10^6^ per well) derived from PF-treated and control EAE mice were cultured in the presence of MOG_35–55_ peptide (20 μg/ml) for 48 h. Culture supernatants were collected and the concentrations of IFN-γ (eBioscience, 88–7314), IL-17 (eBioscience, 88–7371), IL-4 (eBioscience, 88–7044), IL-10 (eBioscience, 88–7105), IL-12 (eBioscience, 88–7121), IL-6 (eBioscience, 88–7064), TGF-β (eBioscience, 88–8350), and TNF-α (eBioscience, 88–7324) were measured by ELISA according to the manufacturer’s instruction. For measuring cytokines secreted by BMDCs, BMDCs were stimulated by LPS (100 ng/ml). Supernatants were collected and evaluated for IL-6, IL-12, and IL-23 (eBioscience, 88–7230) by ELISA.

### Quantitative real-time PCR

Total RNA was extracted by using TRIzol reagent (Invitrogen, 15596026, CA, USA) according to the manufacturer’s instruction. The RNA was reverse transcribed to cDNA using a PrimeScript^TM^ RT reagent kit (TaKaRa, RR037A, Japan). Quantitative real-time PCR was performed using SYBR Premix Ex Taq^TM^ II (TaKaRa, RR420A) and ABI viia 7 real-time PCR system. The PCR conditions were as follows: 95 °C for 30 s, followed by 40 cycles of 95 °C for 3 s and 60 °C for 30 s, and completed with a product dissociation cycle. Results were presented as relative folds to the control group (assigned a value of 1) using the standard 2^−ΔΔCT^ method and β-actin was used as a reference gene. Sequences of PCR primer pairs were as follows: β-actin, forward 5′-TGTCCACCTTCCAGCAGATGT-3′ and reverse 5′-AGCTCAGTAACAGTCCGCCTAG-3′; T-bet, forward 5′-CAGCCTGGGGACGCCCTACT-3′ and reverse 5′-CCGGGAAAGCCAGCCAC CTG-3′; Gata3, forward 5′-CTCGGCCATTCGTACATGGAA-3′ and reverse 5′-GGATACCTCTGC ACCGTAGC-3′; Rora, forward 5′-GCCAAACGCATTGATGGATT-3′ and reverse 5′-GCAC GGCACATCCTAATAAACA-3′; Rorc, forward 5′-GGAGCTCTGCCAGAATGACC-3′ and reverse 5′-CAAGGCTCGAAACAGCTCCAC-3′; IL-6, forward 5′-TTCCATCCAGTTGCCTTC TTG-3′ and reverse 5′-TTGGGAGTGGTATCCTCTGTGA-3′; IL-12p35, forward 5′-GTGTCAAT CACGCTACCTCCTCT-3′ and reverse 5′-CCGTCTTCACCATGTCATCTGT-3′; IL-12/23p40, forward 5′-TGGTTTGCCATCGTTTTGCTG-3′ and reverse 5′-ACAGGTGAGGTTCACTGTT TCT-3′; IL-17, forward 5′-TTTAACTCCCTTGGCGCAAAA-3′ and reverse 5′-CTTTCCCT CCGCATTGACAC-3′.

### Co-culturing naïve CD4^+^ T cells with DCs under Th17-polarizing conditions

Naïve CD4^+^ T cells were co-cultured with CD11c^+^DCs (ratio 10:1) sorted from PF-treated and control EAE mice spleens under Th17-polarizing condition (IL-6, 5 ng/ml; TGF-β, 1 ng/ml; IL-23, 50 ng/ml; anti-IFN-γ, 10 μg/ml; anti-IL-4, 10 μg/ml). For co-culturing with BMDCs, the ratio was 20:1. After 3 days of culture, cells were stained with Abs against IL-17 and IFN-γ or p-STAT3, and then analyzed by flow cytometry as described above.

### Immunoblot analysis

BMDCs with or without PF treatment were stimulated with LPS (100 ng/ml) for 15 min and then harvested and homogenized in ice-cold RIPA lysis buffer containing phosphorylated protease inhibitor cocktail (Roche, 04906845001). Total cell lysates were collected, and equal quantities of protein were separated by SDS-PAGE and blotted onto a PVDF membrane. After 1 h of blocking with 5% nonfat milk at room temperature, the membranes were incubated overnight at 4 °C with specific primary Abs: anti-phospho-Ikkα/β (Cell Signaling Technology, 2697, 16A6, CA, USA), anti-Ikkβ (Cell Signaling Technology, 2370, 2C8), anti-phospho-IκBα (Cell Signaling Technology, 4814, L35A5), anti-IκBα (Cell Signaling Technology, 2859, 14D4), anti-phospho-NF-κB p65 (Cell Signaling Technology, 3033, 93H1), anti-NF-κB p65 (Cell Signaling Technology, 8242, D14E12), anti-phospho-ERK1/2 (Cell Signaling Technology, 4370, D13.14.4E), anti-ERK1/2 (Cell Signaling Technology, 4695, 137F5), anti-phospho-p38 (Cell Signaling Technology, 4511, D3F9), anti-p38 (Cell Signaling Technology, 8690, D137E1), anti-phospho-JNK (Cell Signaling Technology, 4668, 81E11), anti-JNK (Cell Signaling Technology, 9252), anti-β-actin (Sigma-Aldrich, SAB5500001, SP124) and anti-tubulin (Abcam, ab52623, EP1569Y, UK). After washing three times with 1× Tris-buffered saline/Tween-20 buffer, the membranes were incubated with appropriate HRP-conjugated polyclonal IgG Abs for 1 h at room temperature. The protein bands were detected using ECL regents (Thermo, 34094, IL, USA).

### Immunofluorescence assay

BMDCs without or with PF (5 μM) treatment were stimulated with LPS (100 ng/ml) for 30 min. Then cells were fixed with 4% paraformaldehyde for 20 min and further permeabilized with 0.1% TritonX-100 for 5 min. After washing with PBS, cells were collected for preparing cell smears. The smears were blocked with 1% bovine serum albumin for 45 min, treated with anti-NF-κB-p65 antibody (1:400) at 4 °C overnight, followed by Alexa Fluor 555-labeled anti-rabbit IgG (1:1000; Cell Signaling Technology, 4413) incubation. DAPI was used to stain the nuclei. Slides were observed under a fluorescence microscope (Olympus, Tokyo, Japan).

### Statistical analysis

All experiments were repeated for at least three times and data were shown as mean ± SEM. One-way ANOVA was performed to determine whether an overall statistically significant change existed before the Student’s *t*-test was used to analyze the difference between two groups. Differences were considered significant when the P value was < 0.05.

## Additional Information

**How to cite this article:** Zhang, H. *et al*. Paeoniflorin Ameliorates Experimental Autoimmune Encephalomyelitis via Inhibition of Dendritic Cell Function and Th17 Cell Differentiation. *Sci. Rep.* **7**, 41887; doi: 10.1038/srep41887 (2017).

**Publisher's note:** Springer Nature remains neutral with regard to jurisdictional claims in published maps and institutional affiliations.
